# Transcutaneous Administration of Imiquimod Promotes T and B Cell Differentiation into Effector Cells or Plasma Cells

**DOI:** 10.3390/pharmaceutics14020385

**Published:** 2022-02-10

**Authors:** Sachiko Hirobe, Taki Yamasaki, Sayami Ito, Ying-Shu Quan, Fumio Kamiyama, Masashi Tachibana, Naoki Okada

**Affiliations:** 1 Laboratory of Clinical Pharmacology and Therapeutics, Graduate School of Pharmaceutical Sciences, Osaka University, 1-6 Yamadaoka, Suita 565-0871, Osaka, Japan; hirobe-s@phs.osaka-u.ac.jp; 2 Department of Molecular Pharmaceutical Science, Graduate School of Medicine, Osaka University, 2-2 Yamadaoka, Suita 565-0871, Osaka, Japan; 3 Department of Pharmacy, Osaka University Hospital, 2-15 Yamadaoka, Suita 565-0871, Osaka, Japan; 4 Project for Vaccine and Immune Regulation, Graduate School of Pharmaceutical Sciences, Osaka University, 1-6 Yamadaoka, Suita 565-0871, Osaka, Japan; nthngsthsmsmn@gmail.com (T.Y.); q8v383@gmail.com (S.I.); tacci@phs.osaka-u.ac.jp (M.T.); 5 CosMED Pharmaceutical Co., Ltd., 32 Higashikujokawanishi-cho, Minami-ku, Kyoto 601-8014, Kyoto, Japan; quan@cosmed-pharm.co.jp (Y.-S.Q.); kamiyama@cosmed-pharm.co.jp (F.K.); 6 Laboratory of Vaccine and Immune Regulation (BIKEN), Graduate School of Pharmaceutical Sciences, Osaka University, 1-6 Yamadaoka, Suita 565-0871, Osaka, Japan

**Keywords:** transcutaneous immunization, microneedle, TLR7 ligand, adjuvant, imiquimod

## Abstract

We are interested in promoting the development of transcutaneous immunization using microneedle technology and attempting to apply an adjuvant with transcutaneous immunization to improve the efficacy and reduce the amount of antigen and number of administrations needed. In this study, we collected basic information to help elucidate the mechanism responsible for the transcutaneous adjuvant activity of imiquimod (IMQ), which is a ligand of toll-like receptor (TLR) 7. In mouse groups administered ovalbumin (OVA), the OVA-specific IgG antibody titer of the IMQ-adjuvanted group was higher than that of the group administered OVA alone. No immune response bias due to transcutaneous IMQ administration was observed in terms of IgG1 (T helper cell [Th]2-type IgG subclass) and IgG2c (Th1-type IgG subclass) antibody titers. After the initial immunization, the IMQ-adjuvanted group showed increased migration of Langerhans cells to draining lymph nodes (dLNs) and active proliferation of OVA-specific CD4^+^ T cells. Transcutaneously administered IMQ did not affect the direction of CD4^+^ T cell differentiation, while promoted B cell activation and germinal center (GC) B cell differentiation. Immune staining revealed greater GC formation in the dLNs with the IMQ-adjuvanted group than in the OVA-alone group. In the secondary immune response, effector T cells increased in the dLNs and spleen, and effector memory T cells also increased in the spleen in the IMQ-adjuvanted group. In addition, our results suggested that the administration of IMQ enhanced B cell differentiation into plasma cells and GC B cells in the dLNs and spleen. In this study, we partially clarified the mechanism underlying the adjuvant activity of transcutaneously administered IMQ, which is required for the practical application of transcutaneous immunization with IMQ.

## 1. Introduction

Vaccines represent the only radical preventive measures against infectious diseases, and since the world’s first vaccination (smallpox vaccine) was developed by Jenner in 1796, humankind has overcome various infectious diseases by vaccination. However, injectable vaccinations, which account for the majority of current vaccine products, are problematic in that they (1) can be very painful, (2) require medical personnel for administration, and (3) require consistent low-temperature control (cold chain) during transportation and storage. The latter two problems can prevent the spread of vaccination in developing countries and hinder the establishment of a rapid vaccine-supply system during the early stages of an epidemic, which is the most important period for controlling or preventing pandemics. We are interested in promoting research on the practical application of transcutaneous immunization using microneedle (MN) technology [1,2]. MNs less than 1 mm do not reach the nociceptors in the skin (and, thus, do not cause strong pain), and self-administration is expected because applying an MN patch to the skin is easy. Furthermore, because MN patches are prepared from dry materials, it is expected that they can be transported and stored at room temperature and applied as new vaccine preparations that overcome the above problems. The efficacy and safety of transcutaneous immunization using MN patches has already been demonstrated in animal experiments and clinical studies [1,2,3,4]. Based on these results, we are currently interested in applying transcutaneous immunization using MN patches for various infectious diseases.

Most antigens used in current inactivated vaccines have low immunogenicity, and the antigens alone cannot induce sufficient acquired immunity to suppress the onset of diseases and alleviate symptoms. Therefore, an important issue in vaccine development is improving the efficacy, which is often explored by enhancing the immunogenicity of antigens. In addition, cost reduction is desired because preventing infectious diseases with vaccines is expensive, due to the large amount of antigen required for one administration and the frequent requirement for multiple administrations before an effect is obtained. Furthermore, developing vaccination strategies that better control the bias of the immune response in terms of the T helper cell (Th)1/Th2 balance can be expected to expand the number of applications for preventing infectious diseases and for treating various immune diseases. We attempted to apply an adjuvant to transcutaneous immunization as a measure to resolve these issues.

Injectable vaccines currently approved in Japan involve the use of adjuvants such as aluminum salts, squalene emulsions, and monophosphoryl lipids. However, it is difficult to prepare a dry formulation for transcutaneous immunization due to its physicochemical properties, in addition to the reported side effects such as allergic reactions and inflammatory reactions at the administration site. Therefore, we focused on ligands for toll-like receptors (TLRs), which are important for the crosstalk between innate and acquired immunity. TLR ligands can act as adjuvants in transcutaneous administration, have few side effects, and can be prepared as dry formulations. When antigen-presenting cells (APCs) phagocytose pathogens that invade the body, antigens are presented to T cells and B cells. At that time, signals are transmitted by TLRs that recognize pathogen-associated molecular patterns found on pathogens, and the production of cytokine production and co-stimulatory molecules can increase, which promotes the adaptive immune system.

Imiquimod (IMQ), a derivative of a TLR7 ligand, is used in topical formulations in humans. The transcutaneous vaccination system is a new route of administration; it is considered useful when a substance that has been clinically used in humans, such as IMQ, is applied as an adjuvant treatment in early clinical application. Another advantage of IMQ is that the transcutaneously administered preparation is clinically used. However, IMQ can also be used to establish a psoriasis model, so it is necessary to make a proper dose setting for its use as an adjuvant. Although we have previously screened adjuvant candidates, the adjuvant activity of IMQ could not be confirmed. However, the dose setting has been evaluated by only one point of dose. Furthermore, little information is available regarding the mechanism underlying the transcutaneous adjuvant activity of IMQ. Therefore, in the present study, we aimed to (1) validate the usefulness of IMQ for transcutaneous vaccination by determining the setting of dose, and (2) form a scientific basis for efficacy and safety of IMQ by data collection.

## 2. Materials and Methods

### 2.1. Mice

C57BL/6 mice (H-2K^b^, female, 7 weeks old) were purchased from Japan SLC, Inc. (Shizuoka, Japan). OT-II transgenic mice, designated B6.Cg-Tg(TcraTcrb)425Cbn/J mice, and CD45.1-transgenic mice with a B6 background, designated B6.SJL-*Ptprc^a^ Pepc^b^*/BoyJ mice, were purchased from Jackson Laboratory (Bar Harbor, ME). OT-II and CD45.1 mice were bred to produce OT-II/CD45.1 mice. The mice were maintained and bred in an experimental animal facility at Osaka University. All experiments were performed on mice that were 7–12 weeks of age. Throughout this study, all procedures involving laboratory animals were conducted according to the guidelines and policies of the Act on Welfare and Management of Animals in Japan. All protocols and procedures were approved by the Animal Care and Use Committee of Osaka University (protocol number: Douyaku 28-6). Experiments were conducted on animals that were anesthetized by intranasal administration of 2% isoflurane.

### 2.2. Reagents and Antibodies

Ovalbumin (OVA) was purchased from FUJIFILM Wako Pure Chemical Corporation (Osaka, Japan). IMQ was purchased from Nacalai Tesque, Inc. (Kyoto, Japan). The ultra-sensitive TMB substrate was purchased from Moss, Inc. (Pasadena, MD, USA). Horseradish peroxidase (HRP)-conjugated anti-mouse IgG, HRP-conjugated anti-mouse IgG1, HRP-conjugated anti-mouse IgG2c, and fluorescein isothiocyanate (FITC)-conjugated anti-mouse IgG1 were purchased from Southern Biotechnology (Birmingham, AL, USA). Mouse CD4^+^ T cell-isolation kits were purchased from Miltenyi Biotec (Bergisch Gladbach, Germany). Fluorescein anti-peanut agglutinin (PNA) was purchased from Vector Laboratories (Burlingame, CA, USA).

The following were purchased from BioLegend (San Diego, CA, USA): rat anti-mouse CD16/CD32 (93; blocks-Fc binding), Brilliant Violet^TM^ 421-conjugated anti-mouse CD45.1 (A20), phycoerythrin (PE)-conjugated anti-mouse IgD (11-26c.2a), Brilliant Violet^TM^ 421-conjugated anti-mouse/human B220 (RA3-6B2), FITC-conjugated anti-mouse CD19 (6D5), Alexa Fluor^®^647-conjugated anti-mouse/human GL7 (GL7), Pacific Blue-conjugated anti-mouse/rat/human CD27 (LG.3A10), peridinin-chlorophyll-protein (PerCP)-Cy5.5-conjugated anti-mouse CD69 (H1.2F3), Pacific Blue-conjugated anti-mouse MHC class II (I-A^b^) (AF6-120.1), allophycocyanin-conjugated anti-mouse CD86 (GL-1), PE-cyanine 7 (Cy7)-conjugated anti-mouse CD62L (MEL-14), allophycocyanin-conjugated anti-mouse CD127 (A7R34), allophycocyanin-Cy7-conjugated anti-mouse/human CD44 (IM7), allophycocyanin-Cy7-conjugated anti-mouse CD11c (N418), PE-conjugated anti-mouse MHC class II (I-A^b^) (AF6-120.1), Alexa Fluor^®^594-conjugated anti-mouse CD3 (17A2), Alexa Fluor^®^647-conjugated anti-mouse IgD (11-26c.2a), the Zombie Green Fixable Viability Kit, and the Zombie Aqua Fixable Viability Kit.

The following were purchased from Thermo Fisher Scientific (Waltham, MA, USA): cell-proliferation dye eFluor 670, PE-conjugated anti-mouse CD40 (1C10), PerCP-Cy5.5-conjugated anti-mouse CD19 (eBio1D3), PE-conjugated anti-mouse CD122 (TM-b1), eFluor 450-conjugated anti-mouse CD4 (GK1.5), allophycocyanin-conjugated anti-mouse CD103 (2E7), eFluor 450-conjugated anti-mouse CD8α (53-6.7), the ProLong^®^ Gold antifade reagent, and UltraPure DNase/RNase-Free Distilled Water.

The following were purchased from BD Biosciences (San Jose, CA, USA): Streptavidin-HRP, biotin-conjugated anti-mouse IgE (R35-118), allophycocyanin-conjugated anti-mouse CD138 (281-2), PE-Cy7-conjugated anti-mouse CD95 (Jo2), PE-conjugated anti-mouse CD80 (16-10A1), Brilliant Violet^TM^ 421-conjugated anti-mouse CD70 (FR70), PE-conjugated anti-mouse CD86 (GL-1), PerCP-Cy5.5-conjugated anti-mouse B220 (RA3-6B2), and Brilliant Violet^TM^ 510-conjugated anti-mouse Ep-CAM (G8.8).

### 2.3. Preparation of Polyglycolic Acid (PGA)-MN Patches and OVA-Loaded Hydrophilic Gel (HG) Patches

PGA-MN and OVA-loaded HG patches were prepared as previously described [5]. The PGA-MN patches were aseptically fabricated using a microinjection molding machine (FANUC CORPORATION, Oshino, Yamanashi, Japan) at an injection temperature of 240 °C. Each PGA-MN patch had 481 microneedles with a length of 300 μm in a circle with a 1 cm diameter. The HG formulations were prepared as previously described [6]. OVA-loaded HG patches were prepared by dropping 50 μL OVA solution (2 mg/mL in distilled water) onto the HG patches (1.0 × 1.0 cm^2^). The solution was quickly absorbed into the base constituents, whereas OVA (100 μg) remained on the patch surface.

### 2.4. Immunization (Poke and Patch Method)

Immunization was performed as previously described [5]. The hair on the back of each mouse was removed using Epilat (Kracie Holdings, Tokyo, Japan) 48 h before vaccination. A PGA-MN patch was applied to the epilated skin of each mouse, and immediately after that, 5 μL of IMQ solution was dropped onto the punctured skin. Finally, an OVA-loaded HG was applied for 24 h to the area that overlapped the MN-application site (Supplemental Figure S1).

### 2.5. Measuring OVA-Specific Antibody Titers

OVA-specific IgG and IgE antibody titers in sera were determined by performing enzyme-linked immunosorbent assays (ELISAs), as previously described [5]. End-point titers of OVA-specific antibodies were expressed as the reciprocal log_2_ value of the highest dilution that had an absorbance of 0.1, after subtracting the background.

### 2.6. Analysis of CD11c^+^ APC Subsets in Draining Lymph Nodes (dLNs)

To analyze CD11c^+^ APC subsets, dLNs (brachial lymph nodes (LNs) + axillary LNs + inguinal LNs) were collected 24 h after immunization and homogenized into cell suspensions. Each cell suspension was incubated with rat anti-mouse CD16/CD32 to block Fc binding. Surface markers (CD11c, CD8α, B220, Ep-CAM, and CD103) and activation markers (MHC class II, CD80, and CD86) were stained with antigen-specific antibodies, and dead cells were stained with Zombie Green. As shown in Supplemental Figure S2A, CD11c^+^ live cells (Zombie Green^−^) were divided into five subsets: CD8α^+^ dendritic cells (DCs) (CD8α^+^, B220^−^), plasmacytoid DCs (pDCs) (B220^+^), Langerhans cells (LCs) (CD8α^−^, B220^−^, Ep-CAM^+^), CD103^−^ dermal DCs (dDCs) (CD8α^−^, B220^−^, Ep-CAM^−^, CD103^−^), and CD103^+^ dDCs (CD8α^−^, B220^−^, Ep-CAM^−^, CD103^+^). The abundance of each population in the dLNs was analyzed by flow cytometry (FCM). The expression levels of MHC class II, CD80, and CD86 on each APC subset were also analyzed by FCM and expressed as the geometric mean fluorescence intensity (GMFI).

### 2.7. Proliferation Assay for OVA-Specific CD4^+^ T Cells

CD4^+^ T cells from OT-II mice were isolated from the spleen and LNs using an autoMACS Pro Separator and a Mouse CD4^+^ T Cell Isolation Kit (Miltenyi Biotec). The isolated cells were fluorescently labeled with eFluor 670 and then intravenously administered to each mouse at a dose of 3 × 10^6^ cells/300 μL. On the next day, the mice were immunized. At 3 days post-immunization, dLNs were collected and homogenized to prepare a separate cell suspension from each mouse. Each cell suspension was incubated with rat anti-mouse CD16/CD32 to block Fc binding. CD4 and CD45.1 expression were detected by staining with antigen-specific antibodies, dead cells were stained with Zombie Green, and the proliferation of transferred OT-II cells (CD4^+^, CD45.1^+^) among live cells (Zombie Green^−^) was detected by FCM based on the fluorescence intensity of eFluor 670 (Supplemental Figure S2B).

### 2.8. FCM Analysis of T/B Cell Subsets and B Cell Phenotypes

To analyze Th cell subsets, dLNs were collected 2 weeks after the first immunization, and dLNs and spleens were collected 2 weeks after the third immunization at 2-week intervals. They were homogenized into separate cell suspensions, and the cell suspensions were incubated with rat anti-mouse CD16/CD32 to block Fc binding. Effector/memory T cell markers (CD4, CD44, CD122, CD127, and CD62L) were stained with antigen-specific antibodies, and dead cells were stained with Zombie Aqua. As shown in Supplemental Figure S2C, the Th cells (CD4^+^) among the live cells (Zombie Aqua^−^) were classified into five types: naive T (T_N_) cells (CD44^low^, CD122^−^), stem cell memory T (T_SCM_) cells (CD44^low^, CD122^+^), central memory T (T_CM_) cells (CD44^high^, CD127^+^, CD62L^high^), effector memory T (T_EM_) cells (CD44^high^, CD127^+^, CD62L^low^), and effector T (T_EFF_) cells (CD44^high^, CD127^−^, CD62L^low^).

To analyze B cell activation, dLNs were collected 2 weeks after immunization and homogenized into separate cell suspensions. Each cell suspension was incubated with rat anti-mouse CD16/CD32 to block Fc binding. Surface markers (CD19, MHC class II, CD40, CD80, CD86, CD69, CD70, GL7, and CD138) were stained with antigen-specific antibodies, dead cells were stained with Zombie Aqua, and GMFIs were calculated as the expression levels of MHC class II, CD40, CD80, CD86, CD69, CD70, GL7, and CD138 in live (Zombie Aqua^−^) B cells (CD19^+^) by FCM.

To analyze B cell subsets, dLNs were collected 2 weeks after the first immunization, and dLNs and spleens were collected 2 weeks after the third immunization at 2-week intervals. After collection, the samples were homogenized to prepare separate cell suspensions. The cell suspensions were incubated with rat anti-mouse CD16/CD32 to block Fc binding. B cell and plasma cell surface markers (CD19, IgD, GL7, CD95, CD27, B220, and CD138) were stained with antigen-specific antibodies, and dead cells were stained with Zombie Aqua. As shown in Supplemental Figure S2D, live (Zombie Aqua^−^) B cells were classified into four types: naive B cells (CD19^+^, IgD^+^), germinal center (GC) B cells (CD19^+^, IgD^−^, GL7^+^, CD95^+^), memory B cells (CD19^+^, IgD^−^, GL7^−^, CD27^+^), and plasma cells (B220^−^, IgD^−^, CD138^+^). The relative abundances of these cell populations were determined by FCM.

### 2.9. Immunofluorescence Staining of GCs

dLNs were collected 2 weeks after immunization, immersed in phosphate-buffered saline containing 4% paraformaldehyde, and fixed overnight at 4 °C. Sections were sliced to a thickness of 8 µm and stained for PNA, CD3, and IgD after blocking. After an overnight incubation at 4 °C, the samples were washed and sealed with ProLong^®^ Gold antifade reagent. The stained sections were observed under a fluorescence microscope (BZ-8000; Keyence, Osaka, Japan).

### 2.10. Statistical Analyses

The data presented are expressed as the mean ± standard error (SE) of the results obtained from three to six mice. Statistical analysis was performed using Student’s *t*-test or Williams’ test.

## 3. Results

### 3.1. Effect of Combining IMQ Administration with OVA on the OVA Antibody-Production Profile

**Figure 1:**
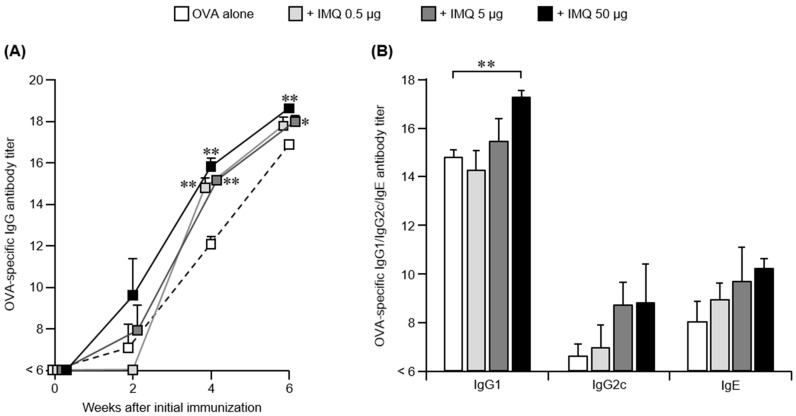
OVA-specific antibody production in combination with IMQ administration. C57BL/6 mice were immunized using the poke and patch method with OVA or OVA plus IMQ (0.5, 5, or 50 μg) three times every 2 weeks. (**A**) Sera collected every 2 weeks were assayed to determine OVA-specific total IgG titers by ELISA analysis. (**B**) Sera collected 2 weeks after the last immunization were assayed to determine IgG subclass titers and IgE titers by ELISA. The data shown are expressed as the mean ± SE of the results from four mice (Williams’ test, * *p* < 0.05, ** *p* < 0.01, versus OVA alone).

First, the dose dependence of IMQ on antigen-specific antibody production was examined. The serum OVA-specific total IgG antibody titer increased slightly after the initial immunization when the mice were subjected to immunization with OVA alone, and the antibody titers increased with successive immunizations (Figure 1A). When IMQ was used in combination with 0.5, 5, or 50 μg, no significant increase was observed with any of the combined doses, compared to OVA immunization alone, after the initial immunization. However, after the second immunization, the OVA-specific IgG antibody titers significantly increased by a 4 (2^2^)-fold increase in log_2_ titer with IMQ co-treatment, and increased in a dose-dependent manner. In addition, even after the third immunization, the IgG antibody titer tended to increase in a dose-dependent manner.

OVA-specific IgG subclass analysis performed with serum samples after the third immunization revealed that co-administering IMQ tended to dose-dependently increase IgG1 (Th2 type subclass) antibody titers, when compared to administering OVA alone (Figure 1B). The IgG2c (Th1 type subclass) antibody titers also tended to increase in a dose-dependent manner, although no significant difference was observed. These results suggest that administering IMQ transcutaneously enhanced antigen-specific IgG antibody production without affecting the Th1/Th2 balance. Furthermore, after the third immunization, the serum OVA-specific IgE antibody titers also tended to increase in a dose-dependent manner, suggesting that transcutaneous IMQ administration also enhanced antigen-specific IgE antibody production.

These results suggest that transcutaneously administering IMQ enhanced antigen-specific antibody production without affecting the immune-response bias.

### 3.2. Migration and Activation of APCs after Combined OVA + IMQ Administration

**Figure 2:**
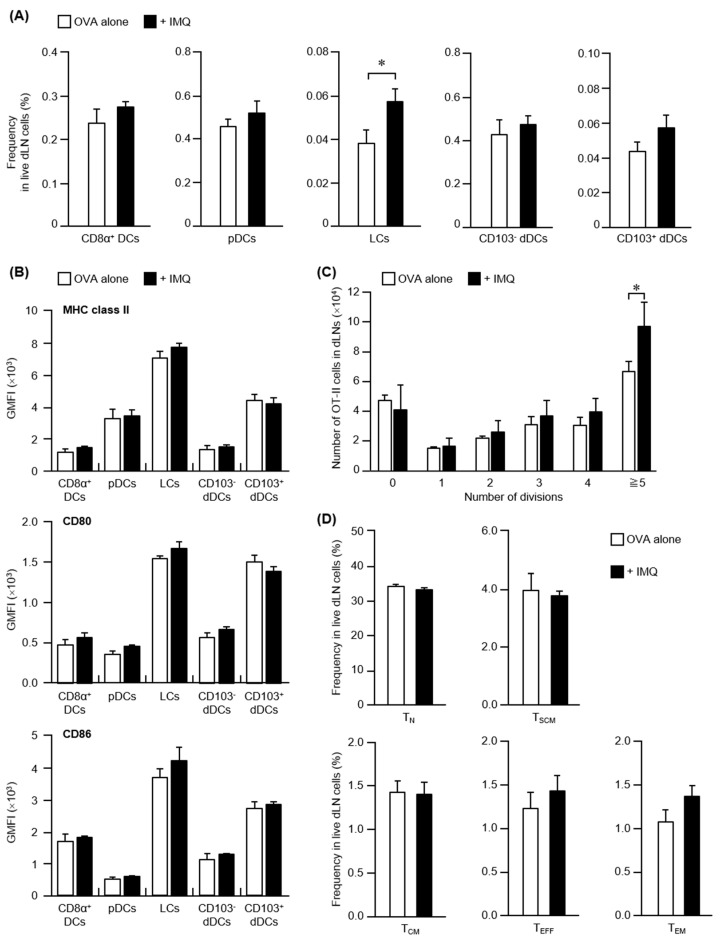
Migration and activation of various APC subsets and proliferation and differentiation of CD4^+^ T cells. (**A**,**B**) C57BL/6 mice were immunized using the poke and patch method with OVA or OVA plus IMQ (50 μg). After 24 h, APC subsets in dLNs were analyzed by FCM. CD11c^+^ cells were divided into five subsets: CD8α^+^ DCs (CD8α^+^, B220^−^), pDCs (B220^+^), LCs (CD8α^−^, B220^−^, Ep-CAM^+^), CD103^−^ dDCs (CD8α^−^, B220^−^, Ep-CAM^−^, CD103^−^), and CD103^+^ dDCs (CD8α^−^, B220^−^, Ep-CAM^−^, CD103^+^). (**A**) The frequency of each population in dLNs was analyzed by FCM. The data shown are expressed as the mean ± SE of the results from six mice (Student’s *t*-test, * *p <* 0.05). (**B**) The expression levels of MHC class II, CD80, and CD86 on each APC subset were analyzed by FCM and are presented as GMFIs. The data shown are expressed as the mean ± SE of the results from six mice. “a.u.” means arbitrary unit. (**C**) C57BL/6 mice (CD45.2^+^) were administered eFluor 670-labeled OT-II cells (CD4^+^, CD45.1^+^). On the next day, these mice were immunized using the poke and patch method with OVA or OVA plus IMQ (50 μg). Three days later, proliferation of the transferred OT-II cells (CD4^+^, CD45.1^+^) in dLNs was analyzed by FCM. The number of divisions was detected by the fluorescence intensity of eFluor 670, and number of OT-II cells are shown for each number of divisions. The data shown are expressed as the mean ± SE of the results from three mice (Student’s *t*-test, * *p <* 0.05). (**D**) C57BL/6 mice were immunized using the poke and patch method with OVA or OVA plus IMQ (50 μg). Two weeks after, differentiation into CD4^+^ T cells in dLNs was analyzed by FCM. CD4^+^ T cells were divided into five subsets: T_N_ cells (CD44^low^, CD122^−^), T_SCM_ cells (CD44^low^, CD122^+^), T_CM_ cells (CD44^high^, CD127^+^, CD62L^high^), T_EM_ cells (CD44^high^, CD127^+^, CD62L^low^), and T_EFF_ cells (CD44^high^, CD127^−^, CD62L^low^). The frequency of each population in dLNs was analyzed by FCM. The data shown are expressed as the mean ± SE of the results from five to six mice.

The possibility that enhanced antigen presentation contributed to transcutaneous adjuvant activity of IMQ was examined. A subset analysis of APCs in dLNs was performed 24 h after immunization. APCs were divided into CD8α^+^ DCs, pDCs, LCs, CD103^–^ dDCs, and CD103^+^ dDCs by staining for cell surface markers (Supplemental Figure S2A). When the abundances of each subset in dLNs were compared between the OVA group and the OVA + IMQ-combination group, no significant changes were observed in terms of the abundances of LN-resident CD8α^+^ DCs and pDCs, or dermis-resident CD103^–^ dDCs and CD103^+^ dDCs (Figure 2A). In contrast, the abundance of epidermis-resident LCs increased significantly in the IMQ-combination group compared to the OVA-alone group. These results suggest that the combined use of IMQ increased LC migration to dLNs.

Furthermore, we considered that transcutaneous IMQ administration may affect not only the migration of LCs to dLNs but also their activation state after migration to dLNs. Therefore, we analyzed the expression levels of MHC class II (which is responsible for antigen presentation) and CD80 and CD86 (which are co-stimulatory molecules). In the OVA-alone group, LCs showed the highest MHC class II expression among the APC subsets in dLNs (Figure 2B), suggesting that LCs had the highest potential for antigen presentation during the immune response induced by transcutaneous immunization. However, the expression intensity was the same with IMQ co-administration, suggesting that transcutaneously administering IMQ did not affect the antigen-presenting abilities of LCs that migrated to dLNs. In addition, LCs showed the highest CD80 and CD86 expression intensities among the APC subsets in dLNs after transcutaneous immunization with OVA alone, and similar expression levels were observed when IMQ was included. These results suggest that transcutaneously administering IMQ did not affect the expression levels of co-stimulatory molecules in LCs that migrated to dLNs.

The above results suggest that the increased migration of LCs to dLNs contributed significantly to the transcutaneous adjuvant activity of IMQ.

### 3.3. Proliferation and Differentiation of CD4^+^ T Cells with Combined OVA + IMQ Administration

LC migration to dLNs increased with the combined use of IMQ (Figure 2A), raising the possibility that enhanced antigen presentation to Th cells and the antigen-specific proliferation of Th cells contributed to the high production of antigen-specific antibodies. We analyzed the division and proliferation of OT-II cells in the dLNs 3 days after immunization (Supplemental Figure S2B). In the OVA-alone group, OT-II cells that had divided and proliferated five times or more were detected, suggesting that antigen-specific Th cells in dLNs proliferated actively after transcutaneous immunization (Figure 2C). Furthermore, in the IMQ-combination group, the number of OT-II cells that divided and proliferated five times or more increased significantly. These results suggest that enhanced antigen-specific proliferation of Th cells after LC migration to dLNs may be one of the effects of including IMQ during transcutaneous immunization.

Since the combined use of IMQ promoted antigen-specific proliferation of Th cells in dLNs (Figure 2C), T cell subset analysis was performed to investigate Th cell differentiation at 2 weeks after the initial immunization. Depending on the stage of differentiation, CD4^+^ Th cells were divided into T_N_, T_SCM_, T_CM_, T_EM_, and T_EFF_ cells [7] (Supplemental Figure S2C). It has been reported that when T_N_ cells receive antigen presentation from APCs, they differentiate in the order of T_SCM_, T_CM_, T_EM_, and T_EFF_ cells, and that T_SCM_ cells can directly differentiate into T_EM_ cells and T_EFF_ cells without passing through T_CM_ cells [8]. When the frequency of each subset in dLNs was compared between the OVA-alone group and the IMQ-combination group, no significant difference was observed for any of the subsets (Figure 2D). These findings suggest that transcutaneous IMQ administration promoted antigen-specific Th cell proliferation during the primary immune response, without affecting the direction of their differentiation.

### 3.4. Activation and Differentiation of B Cells in IMQ Combination

**Figure 3:**
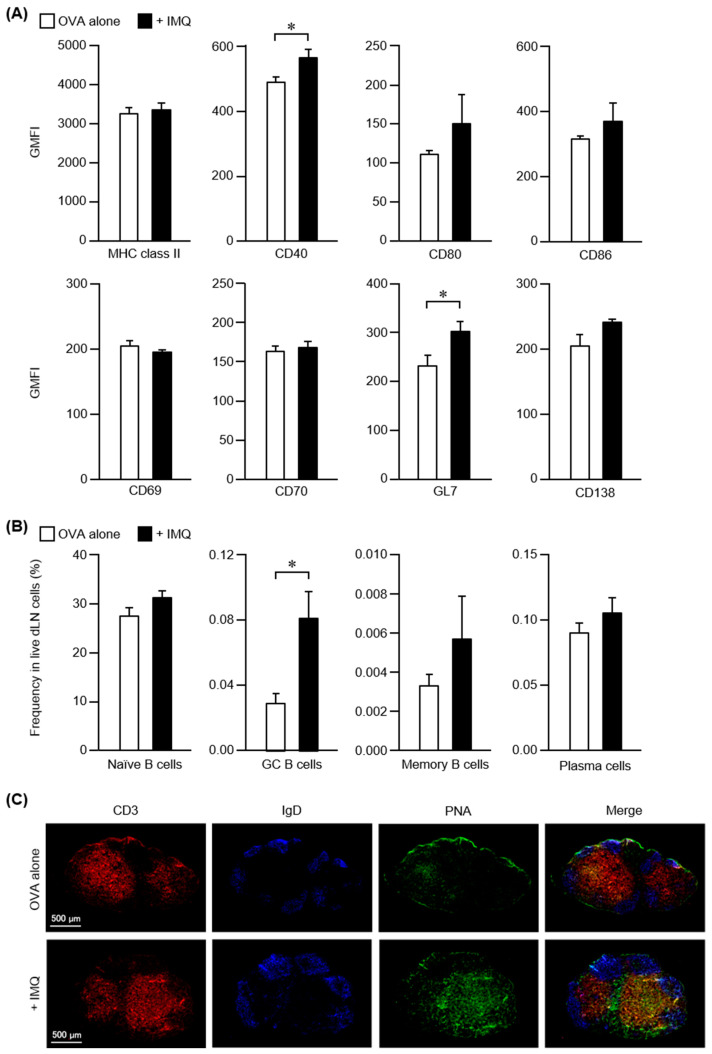
B cell activation and differentiation. C57BL/6 mice were immunized using the poke and patch method with OVA or OVA plus IMQ (50 μg). Two weeks later, B cell activation and differentiation in the dLNs were analyzed by FCM. (**A**) The expression levels of MHC class II, CD40, CD80, CD86, CD69, CD70, GL7, and CD138 on B cells were analyzed by FCM and are presented as GMFIs. The data shown are expressed as the mean ± SE of the results from five (CD70, CD80, CD86, and GL7) or six (MHC class II, CD40, CD69, and CD138) mice (Welch’s *t*-test, * *p <* 0.05). “a.u.” means arbitrary unit. (**B**) B cells were divided into four subsets: naive B cells (CD19^+^, IgD^+^), GC B cells (CD19^+^, IgD^−^, GL7^+^, CD95^+^), memory B cells (CD19^+^, IgD^−^, GL7^−^, CD27^+^), and plasma cells (B220^−^, IgD^−^, CD138^+^). The frequency of each population in the dLNs was analyzed by FCM. The data shown are expressed as the mean ± SE of the results from to five (naive, GC, and memory B cells) or six (plasma cells) mice (Welch’s *t*-test, * *p <* 0.05). (**C**) C57BL/6 mice were immunized using the poke and patch method with OVA or OVA plus IMQ (50 μg). Two weeks later, dLNs were collected, and GCs were stained with antibodies against IgD (blue), CD3 (red), or PNA (green).

Although transcutaneous IMQ administration did not affect the direction of CD4^+^ T cell differentiation (Figure 2D), we speculated that IMQ may affect the activation state and differentiation of B cells. To investigate the effect of co-administering IMQ with OVA on B cell activation, we analyzed the expression intensity of B cell activation markers in dLNs at 2 weeks after the primary immune response. MHC class II is responsible for antigen presentation; CD40, CD80, and CD86 are co-stimulatory molecules; CD69 is an early activation marker [9]; CD70 contributes to IgG production [10]; GL7 is a GC B cell marker [11]; CD138 is a plasma cell marker [12]. When these expression intensities of these proteins were compared between the OVA-alone group and the IMQ-combination group, no significant differences were observed for MHC class II, CD80, CD86, CD69, CD70, and CD138, but the IMQ-combination group showed significantly higher expression of CD40 and GL7 (Figure 3A). These findings suggest that the combined use of IMQ enhanced B cell activation and differentiation into GC B cells.

Subsequently, to investigate the effect of the combined use of IMQ on B cell differentiation, B cell subset analysis in dLNs was performed 2 weeks after the primary immune response. The B cell subsets were fractionated into naive B cells, GC B cells, memory B cells, and plasma cells (Supplemental Figure S2D) [13]. When the frequency of each subset in dLNs was compared between the OVA-alone group and the IMQ-combination group, no significant differences were observed for naive B cells, memory B cells, and plasma cells. However, the frequency of GC B cells in the IMQ-combination group was approximately twice that of the OVA-alone group (Figure 3B). These data indicated that the combined use of IMQ enhanced the B cell differentiation into GC B cells.

The above results suggested that B cells activation and the enhanced differentiation into GC B cells during the primary immune response contributed to the adjuvant activity of IMQ in transcutaneous immunization.

Since the combined use of IMQ enhanced the activation of B cells and their differentiation into GC B cells (Figure 3A,B), we speculated that GC formation was enhanced. GCs in dLNs were observed after the initial immunization by detecting T cells (CD3), naive B cells (IgD), and GC B cells (PNA). In the IMQ-combination group, no clear difference was observed in the T cell and B cell regions, as compared with the OVA-alone group, but GCs were more widely observed in the IMQ-combination group than in the OVA-alone group. Therefore, our results showed that transcutaneous IMQ administration increased LC migration into dLNs during the primary immune response, and then induced rapid antigen-specific Th cell proliferation and GC formation in dLNs, thereby enhancing antigen-specific antibody production.

### 3.5. Differentiation of T and B Cells after Multiple Immunizations with OVA and IMQ

The transcutaneous adjuvant activity of IMQ was stronger during the secondary immune response than in the primary immune response (Figure 1A). Therefore, it was necessary to analyze the effect of IMQ on the secondary immune response to elucidate the mechanism of the adjuvant activity. To this end, we performed a subset analysis of CD4^+^ T cells and B cells in the dLNs and spleen after three immunizations.

**Figure 4:**
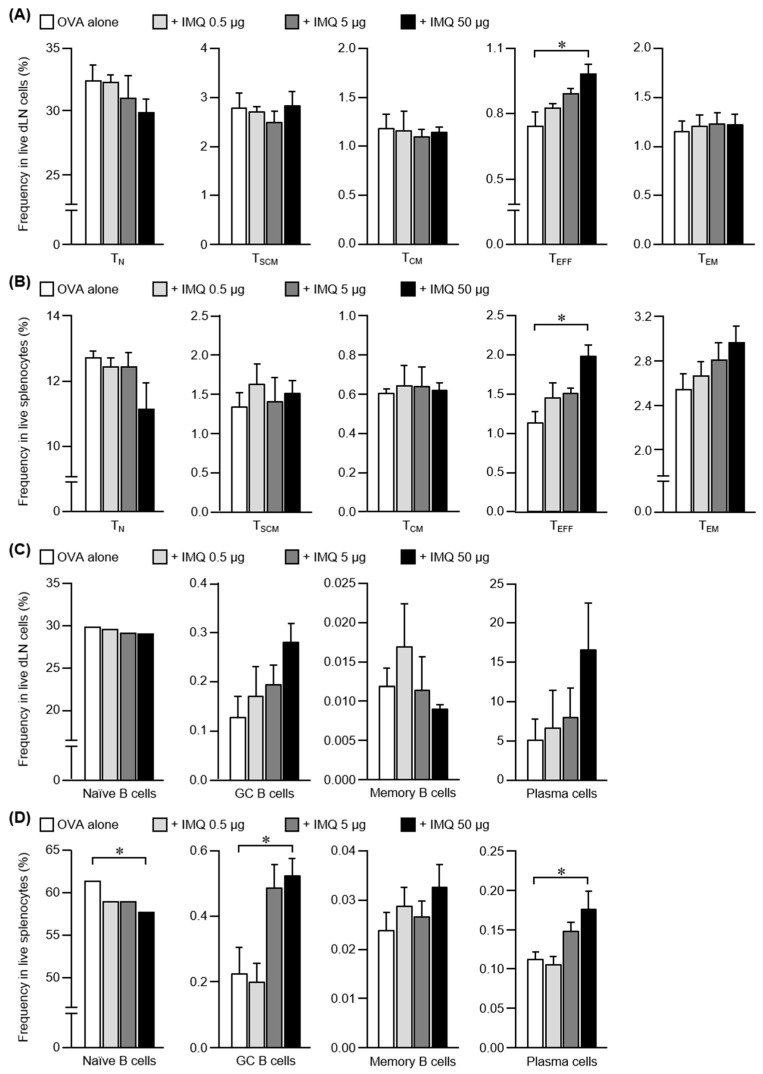
Differentiation of T cells and B cells after multiple immunizations with IMQ. C57BL/6 mice were immunized using the poke and patch method with OVA or OVA plus IMQ (0.5, 5, and 50 μg) three times every 2 weeks. Two weeks after the last immunization, CD4^+^ T cells were divided into five subsets: T_N_ cells, T_SCM_ cells, T_CM_, T_EM_, and T_EFF_ cells. B cells were divided into four subsets: naive B cells, GC B cells, memory B cells, and plasma cells. The frequency of each population in the dLNs (**A**,**C**) or spleen (**B**,**D**) was analyzed by FCM. The data shown are expressed as the mean ± SE of the results from three to four (Williams’ test, * *p <* 0.05, versus OVA alone).

Subset analysis of CD4^+^ T cells showed that the abundance of T_EFF_ cells in dLNs increased in a dose-dependent manner (Figure 4A) and that T_EFF_ cells and T_EM_ cells in the spleen tended to increase (Figure 4B). B cell subset analysis showed that the abundances of GC B cells and plasma cells in dLNs increased in response to IMQ in a dose-dependent increase (Figure 4C). A similar profile was observed in the spleen (Figure 4D). Therefore, our findings suggest that during the secondary immune response, the combined use of IMQ increased the abundances of T_EFF_ cells in dLNs, and T_EFF_ cells and T_EM_ cells in the spleen, which enhanced the GC reaction and differentiation into plasma cells, resulting in more efficient antigen-specific antibody production.

## 4. Discussion

Our research group previously screened various TLR ligands as adjuvants for transcutaneous immunization [5] and evaluated the adjuvant activity of IMQ at doses as high as 100 µg. In that study design, the maximum applicable dose was used for screening purposes; however, it is plausible that proper adjuvant activity in transcutaneous immunity was not observed probably because of excessive immune response and cytotoxicity. In the current study, reducing the dose of IMQ confirmed viable adjuvant activity also in a dose-dependent manner. These results indicate that appropriate dose setting in transcutaneous immunization is required for each candidate adjuvant substance. However, the amount of IMQ delivered into the skin in this method has not yet been evaluated. It is necessary to evaluate the amount of IMQ into the skin in order to set an appropriate dose, and we are considering the evaluation by a method using mass spectrometric imaging in the future. We also conducted evaluations using HG loaded with antigens and IMQ, which confirmed the possibility of enhancing antibody production (Supplemental Figure S3). In this study, we used the poke and patch method because it has a better delivery efficiency to the skin compared to that of HG application and adjuvant activity of IMQ was obtained with a low dose.

Other research groups have conducted studies using IMQ as an ointment, and the application of IMQ to the subcutaneous injection site enhances Th1-type humoral immunity by producing a large amount of Th1-type cytokines locally in the skin [14]. Based on this information, we predicted that the Th1-type immune response was induced when IMQ was used in combination with transcutaneous immunization, although the serum antigen-specific IgG subclass antibody titer did not affect the Th1/Th2 balance in this study. Although a preliminary study, Th1/Th2, Treg, and Th17 levels were evaluated in the dLNs using FCM, and no difference was observed. One factor that may explain these findings is puncturing by MNs. In our previous studies, it was clarified that the expression of genes encoding Th2-type cytokines involved in wound healing (such as IL-10 and TSLP) increased in the skin where the MNs were applied. Thus, we inferred that when performing transcutaneous immunization combined with IMQ administration, the induction of Th1-type cytokines by IMQ and the induction of Th2-type cytokines by MN application antagonize each other locally in the skin. In addition, since it has been reported that LC contributes to the Th2-type immune response after transcutaneous immunization [15], the enhanced migration of LCs may be another factor that hinders the induction of Th1-type immune responses.

TLR7 is expressed in the endosomes of immunocompetent cells such as DCs, monocytes, macrophages, and B cells, and TLR7 signals can promote the expression of various molecules such as cytokines and co-stimulatory molecules [16]. In this study, we clarified that IMQ combination with transcutaneous immunization increased LC migration to dLNs. Therefore, it is necessary to investigate the possibility that IMQ acts directly on LCs to promote the expression of molecules involved in cell migration, such as CCR7. However, previous data have shown that LCs express slightly lower levels of TLR7 than dDCs [5], and it is unlikely that LCs are more sensitive to IMQ than other skin-resident APC subsets. Thus, we inferred that one factor that may explain why IMQ specifically increased LC migration to dLNs is that IMQ is more easily delivered to the epidermis layer than the dermis layer. In the future, an approach to analyze the intradermal dynamics of transcutaneously administered IMQ is desired.

In this study, the number of LCs in dLNs increased, and the proliferation of antigen-specific Th cells was enhanced by the combined use of IMQ with transcutaneous immunization. However, we did not investigate the effect of the combined use of IMQ on the antigen-presentation efficiency of LCs. It is necessary to analyze the presentation efficiency or examine the possibility that LCs deliver antigens to other APC subsets. In addition, LC can cross-present with CD8^+^ T cells [17], and we plan to investigate cell-mediated immunity induced by transcutaneous IMQ administration using transgenic mice that have Langerin- or CD11c-positive cells removed through diphtheria toxin.

The combined use of IMQ enhanced GC formation in dLNs during the primary immune response. In GCs, the interaction between GC B cells and follicular Th (T_FH_) cells results in antibody affinity maturation and class switching [18]. Antibody class switching is induced by the co-stimulatory molecule CD40 on B cells [19], and our results suggested that increased CD40 expression in B cells, induced by combined administration with IMQ and OVA, helped promote GC responses. In the future, we would like to analyze the differentiation of CD4^+^ T cells to T_FH_ cells, and investigate the enhancement of GC responses by IMQ combination with transcutaneous immunization.

Co-administering IMQ did not increase the abundance of T_EFF_ cells in dLNs during the primary immune response, but IMQ increased T_EFF_ cell levels during the secondary immune response. The results may reflect the fact that antigen-specific memory cells remained in the dLNs after the primary immune response. The differentiation into memory cells was not confirmed in the CD4^+^ T cell population as a whole. However, it remains possible that differentiation of the antigen-specific CD4^+^ T cells to memory cells was enhanced. Therefore, it is necessary to analyze the differentiation of antigen-specific T cells to elucidate the mechanism of the transcutaneous adjuvant activity of IMQ.

IMQ has been used as an ointment for treating condyloma acuminata and solar keratosis, and LC migration to dLNs increase even when applied as an ointment [20]. Since it is speculated that IL-1β derived from TLR7-stimulated mast cells contributes to the migration of LCs to dLNs [21], it is necessary to examine the contribution of mast cells to the transcutaneous adjuvant activity of IMQ. Alternatively, intradermal TLR7-expressing cells other than LCs and mast cells may contribute largely to the transcutaneous adjuvant activity of IMQ. It also remains possible that the IMQ that has flowed into the dLNs acts directly on B cells. Additionally, environmental changes in the skin have not been evaluated in the present study, warranting further studies to ensure both efficacy and safety of IMQ. We believe that TLR7 conditional-knockout mice and genetically modified mice with depletion of specific cell types can be used to identify immunocompetent cells that directly contribute to the adjuvant activity of transcutaneously administered IMQ.

Since IMQ has already been used with humans, it is considered that the hurdle to its practical use as an adjuvant for transcutaneous immunization is lower than that of new substances. In addition, the low molecular weight of IMQ offers big advantages in terms of rapid and low-cost manufacturing. Considering the mechanism underlying the transcutaneous adjuvant activity of IMQ suggested in this study, improving the efficacy of transcutaneous immunization can be realized, not only by enhancing acquired immune responses, but also by prolonging immunological memory. In addition, the antigen-specific total IgG antibody titers were comparable between the OVA-alone group after three immunizations and the combination group, after two immunizations with 50 μg IMQ. These findings suggest the possibility of cost reduction. However, since the combined use of IMQ with transcutaneous immunization enhanced antigen-specific antibody production without biasing the immune response, expanding the indication to treat various immune diseases may be difficult. In addition, since both the antigen-specific IgG antibody titer and the IgE antibody titer were slightly increased by the combined use of IMQ, further safety information must be collected before applying IMQ-loaded transcutaneous immunization to patients with allergic diseases. The limitation of this study is that, as mentioned at the beginning of the discussion, there is insufficient research on the amount of IMQ delivered into the skin. In addition, the involvement of APCs and the evaluation of T/B cell activation/differentiation are insufficient, and more detailed studies are required. IMQ is also used to create psoriasis models, although it has already been clinically applied. When used as an adjuvant for vaccination, frequent administration is not expected, but it is also required to evaluate tissue-resident memory T cells from the viewpoint of safety evaluation [22]. In the future, based on the information obtained in this study, we aim to elucidate the mechanism underlying the transcutaneous adjuvant activity of IMQ, and we would like to proceed with studies of practical applications.

## 5. Conclusions

This study suggest that the following factors contribute to the adjuvant activity of IMQ in transcutaneous immunization (Supplemental Figure S4). LC migration to dLNs increased during the primary immune response. These LCs may have promoted antigen-specific Th cell proliferation and GC formation, followed by an increase of T_EFF_ cells in dLNs and T_EFF_ and T_EM_ cells in the spleen. In addition, the secondary immune response enhanced plasma cell differentiation.

## Data Availability

Not applicable.

## Supplementary Materials

The following supporting information can be downloaded at: https://www.mdpi.com/article/10.3390/pharmaceutics14020385/s1, Figure S1: Poke and patch method; Figure S2: Gating and analyzing strategy using flow cytometry; Figure S3: OVA-specific antibody production using HG loaded with OVA and IMQ; Figure S4: Mechanism of the adjuvant activity of IMQ after transcutaneous vaccination.

## Abbreviations

APC	antigen-presenting cell
Cy7	cyanine 7
DC	dendritic cell
dDCS	dermal DC
dLN	draining lymph node
ELISA	enzyme-linked immunosorbent assay
FCM	flow cytometry
FITC	fluorescein isothiocyanate
GC	germinal center
GMFI	geometric mean fluorescence intensity
HG	hydrophilic gel
HRP	horseradish peroxidase
IMQ	imiquimod
LC	Langerhans cell
MN	microneedle
OVA	ovalbumin
pDC	plasmacytoid DC
PE	phycoerythrin
PerCP	peridinin-chlorophyll-protein
PGA	polyglycolic acid
PNA	peanut agglutinin
SE	standard error
TEFF	effector T
TFH	follicular Th
TEM	effector memory T
Th	T helper
TCM	central memory T
TN	naive T
TLR	toll-like receptor
TSCM	stem cell memory T
