# Supplementary material for: Transcutaneous Administration of Imiquimod Promotes T and B Cell Differentiation into Effector Cells or Plasma Cells
Source: Pharmaceutics. 2022 Feb 10;14(2):385. doi: 10.3390/pharmaceutics14020385 (PMC8878978; doi:10.3390/pharmaceutics14020385)
Supplement: Supplementary file 1 [file pharmaceutics-14-00385-s001.zip › pharmaceutics-1587345-supplementary.pdf]

pharmaceutics MDPI

Supplementary Materials: Transcutaneous Administration of Imiquimod Promotes T and B Cell Differentiation into Effector Cells or Plasma Cells

Sachiko Hirobe, Taki Yamasaki, Sayami Ito, Ying-Shu Quan, Fumio Kamiyama, Masashi Tachibana and Naoki Okada

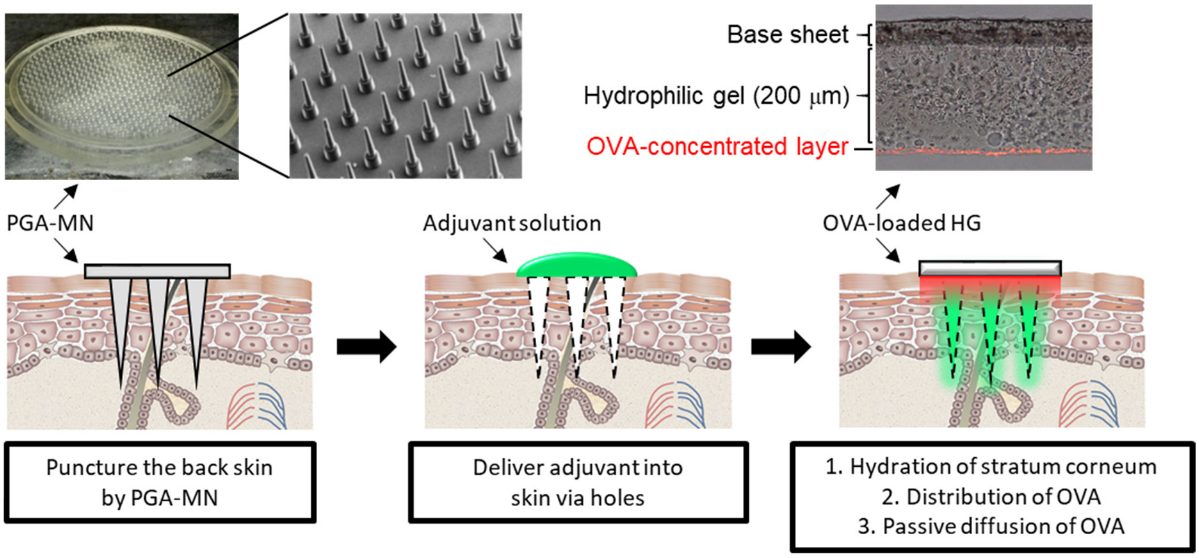

Figure S1. **Poke and patch method.**

The PGA-MN patch was applied to the epilated back skin of each mouse. Then, the adjuvant solution was dropped into the holes formed using PGA-MNs, and OVA-loaded HG was applied to the holes.

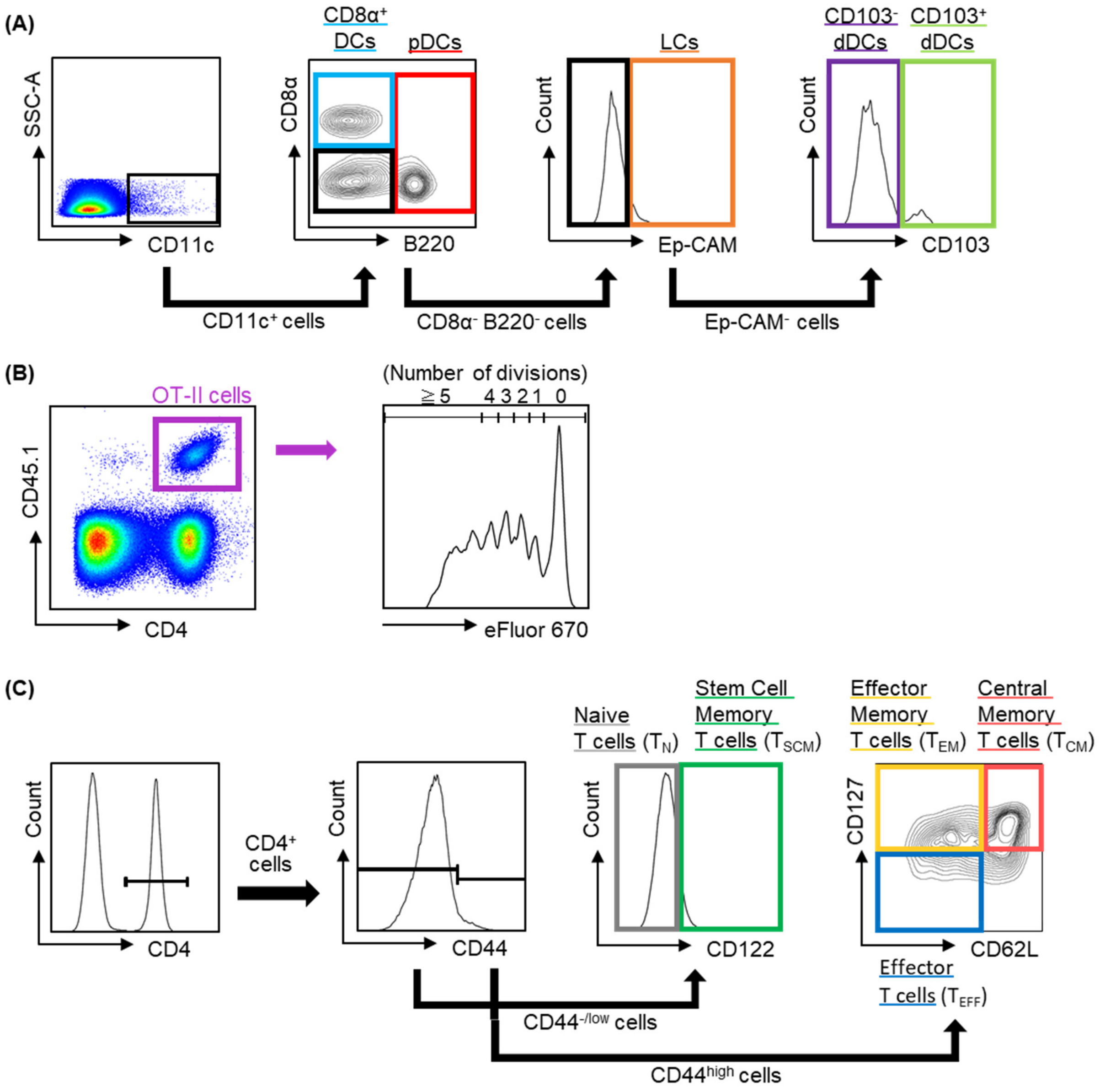
(A)
CD8α+ DCs
pDCs
LCs
CD103- dDCs
CD103+ dDCs
SSC-A
CD11c
CD8α
B220
Count
Ep-CAM
Count
CD103
CD11c+ cells
CD8α- B220- cells
Ep-CAM- cells
(B)
OT-II cells
CD45.1
CD4
(Number of divisions)
≧5 4 3 2 1 0
eFluor 670
(C)
Naive T cells (TN)
Stem Cell Memory T cells (TSCM)
Effector Memory T cells (TEM)
Central Memory T cells (TCM)
Count
CD4
CD4+ cells
Count
CD44
Count
CD122
CD127
CD62L
Effector T cells (TEFF)
CD44-/low cells
CD44high cells

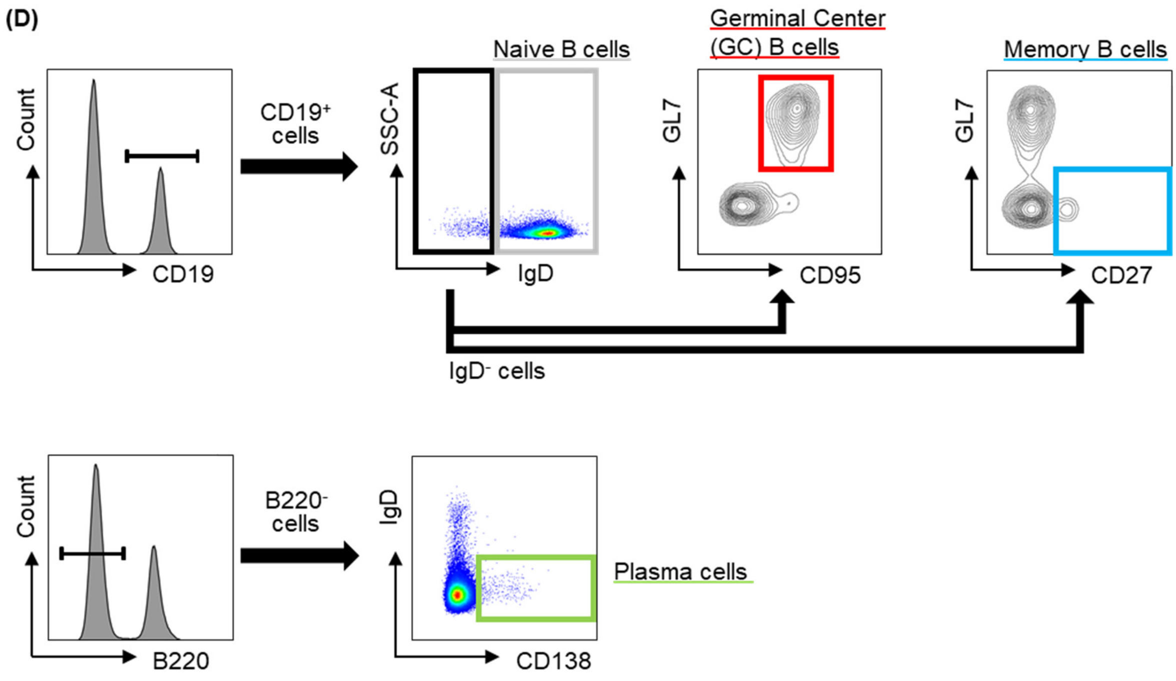

Figure S2. Gating and analyzing strategy using flow cytometry.

(A) $CD11c^+$ cells were divided into five subsets: $CD8\alpha^+$ DCs ($CD8\alpha^+$, $B220^-$), pDCs ($B220^+$), LCs ($CD8\alpha^-$, $B220^-$, Ep-CAM^+), $CD103^-$ dDCs ($CD8\alpha^-$, $B220^-$, Ep-CAM^-, $CD103^-$), and $CD103^+$ dDCs ($CD8\alpha^-$, $B220^-$, Ep-CAM^-, $CD103^+$). (B) C57BL/6 mice ($CD45.2^+$) were administered eFluor 670-labeled OT-II cells ($CD4^+$, $CD45.1^+$). On the next day, the mice were immunized with OVA. Three days later, the proliferation of the transferred OT-II cells ($CD4^+$, $CD45.1^+$) in the dLNs was analyzed by FCM. The number of divisions was determined based on the fluorescence intensity of eFluor 670, and the number of OT-II cells is shown for each number of divisions. (C) $CD4^+$ T cells were divided into five subsets: T_N cells ($CD44^{low}$, $CD122^-$), T_{SCM} cells ($CD44^{low}$, $CD122^+$), T_{CM} cells ($CD44^{high}$, $CD127^+$, $CD62L^{high}$), T_{EM} cells ($CD44^{high}$, $CD127^+$, $CD62L^{low}$), and T_{EFF} cells ($CD44^{high}$, $CD127^-$, $CD62L^{low}$). (D) B cells were divided into four subsets: naive B cells ($CD19^+$, IgD^+), GC B cells ($CD19^+$, IgD^-, $GL7^+$, $CD95^+$), memory B cells ($CD19^+$, IgD^-, $GL7^-$, $CD27^+$), and plasma cells ($B220^-$, IgD^-, $CD138^+$).

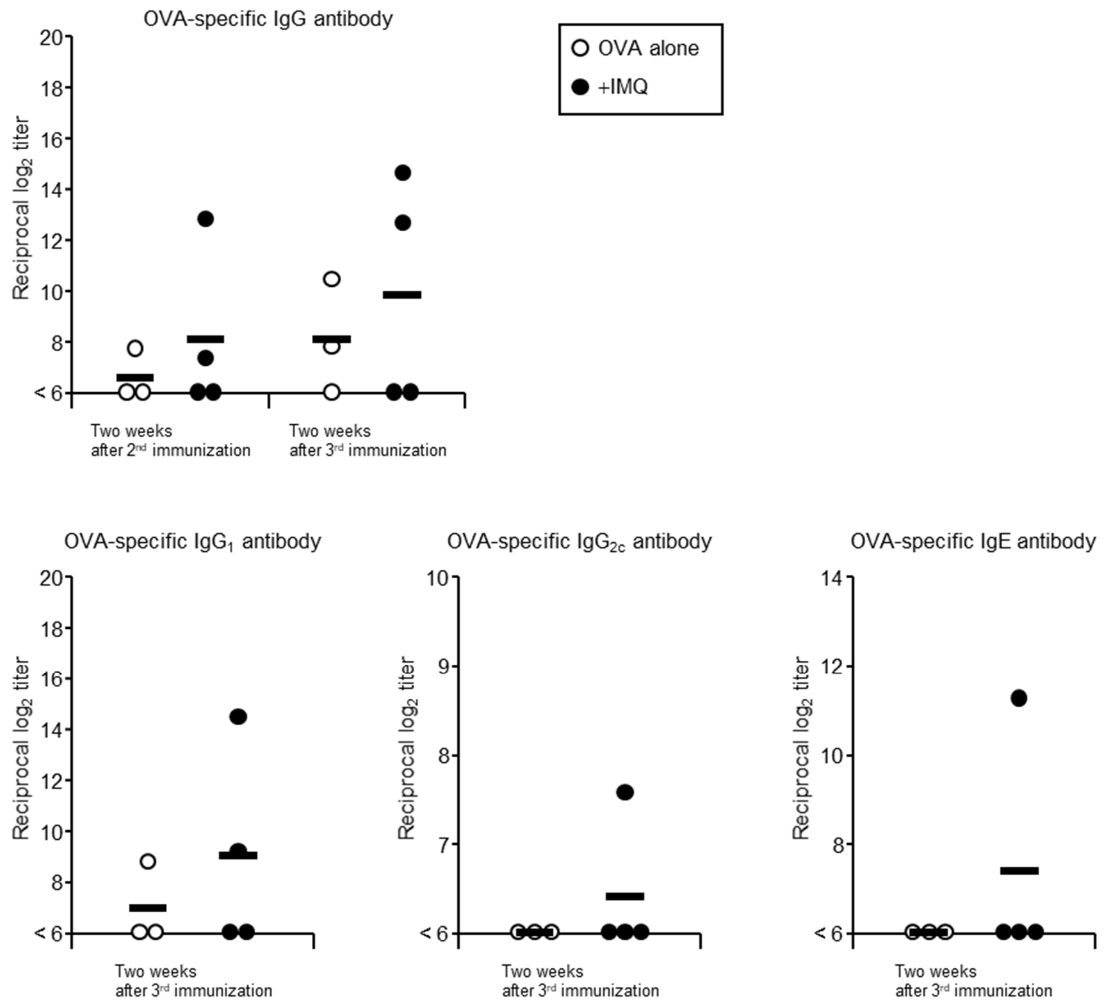

Figure S3. OVA-specific antibody production using HG loaded with OVA and IMQ.

HG loaded with OVA (50 µg) alone or with OVA and IMQ (50 µg) were applied to C57BL/6 mice three times every 2 weeks. Sera collected every 2 weeks were assayed to determine OVA-specific total IgG titers, IgG subclass titers, and IgE titers by ELISA analysis.

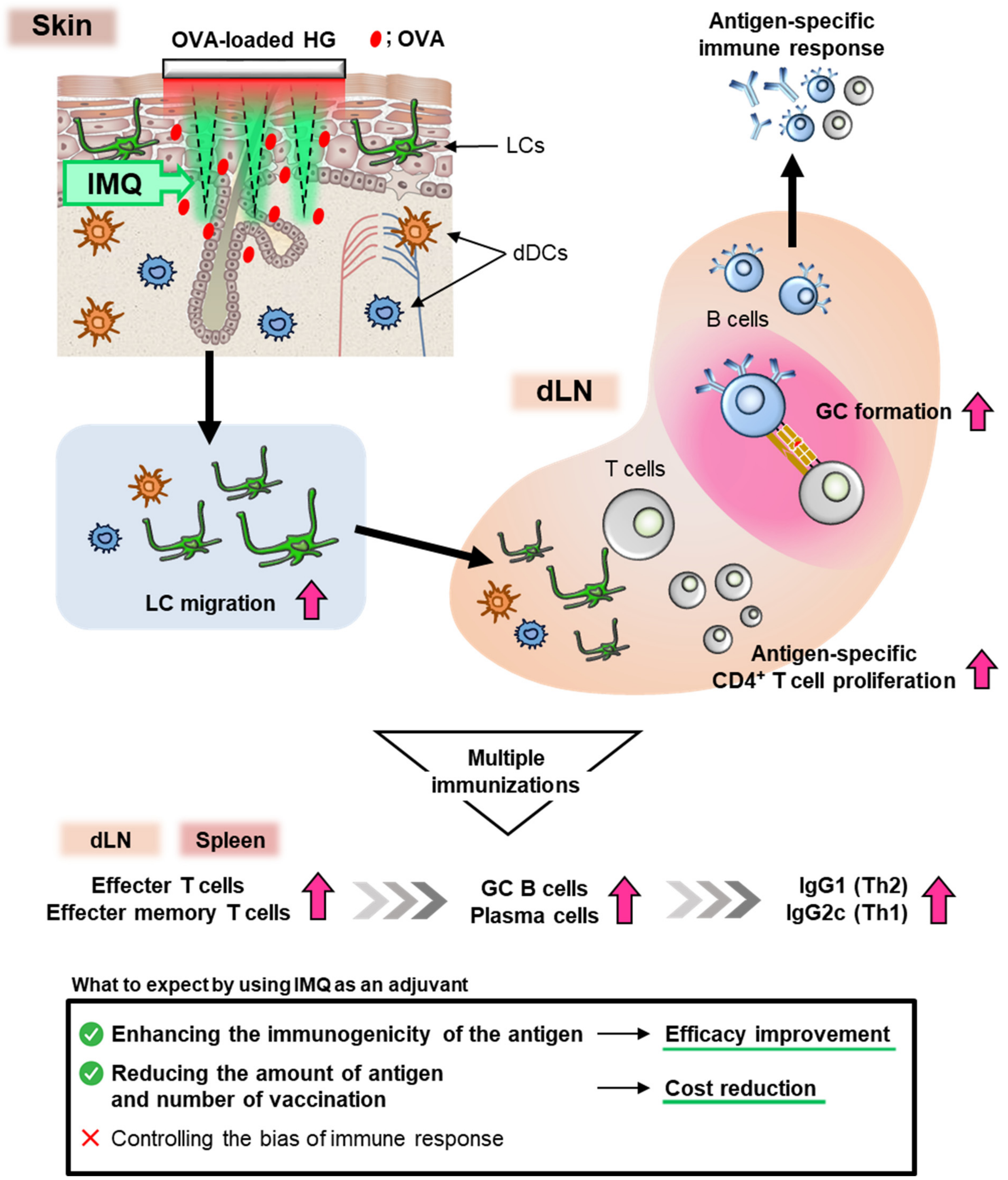

Figure S4. Mechanism of the adjuvant activity of IMQ after transcutaneous vaccination.
Transcutaneously administered IMQ enhances antigen-specific primary immune responses by promoting LC migration, antigen-specific CD4+ T cell proliferation, and GC formation. Furthermore, when performing multiple immunizations, IMQ systemically increased the abundance of TEM cells to enhance TEFF cell responses, promote GC responses, and trigger differentiation into plasma cells. However, IMQ induces IgG1 antibodies (Th2 type IgG subclass) and IgG2c antibodies (Th1 type IgG subclass) in parallel. Accordingly, by using IMQ as an adjuvant in combination with transcutaneous vaccination, we can expect efficacy improvement and cost reduction, but not versatility expansion.
